# Nuclease Tudor-SN Is Involved in Tick dsRNA-Mediated RNA Interference and Feeding but Not in Defense against Flaviviral or *Anaplasma phagocytophilum* Rickettsial Infection

**DOI:** 10.1371/journal.pone.0133038

**Published:** 2015-07-17

**Authors:** Nieves Ayllón, Victoria Naranjo, Ondrej Hajdušek, Margarita Villar, Ruth C. Galindo, Katherine M. Kocan, Pilar Alberdi, Radek Šíma, Alejandro Cabezas-Cruz, Claudia Rückert, Lesley Bell-Sakyi, Mária Kazimírová, Sabína Havlíková, Boris Klempa, Petr Kopáček, José de la Fuente

**Affiliations:** 1 SaBio, Instituto de Investigación en Recursos Cinegéticos IREC, CSIC-UCLM-JCCM, Ronda de Toledo s/n, 13005, Ciudad Real, Spain; 2 Department of Veterinary Pathobiology, Center for Veterinary Health Sciences, Oklahoma State University, Stillwater, Oklahoma, United States of America; 3 Institute of Parasitology, Biology Centre, Academy of Sciences of the Czech Republic, Branišovská 31, 37005, České Budějovice, The Czech Republic; 4 Center for Infection and Immunity of Lille (CIIL), INSERM U1019 –CNRS UMR 8204, Université Lille Nord de France, Institut Pasteur de Lille, Lille, France; 5 The Pirbright Institute, Ash Road, Pirbright, Woking, GU24 0NF, United Kingdom; 6 Institute of Zoology, Slovak Academy of Sciences, Dúbravská cesta 9, 84506, Bratislava, Slovakia; 7 Institute of Virology, Slovak Academy of Sciences, Dúbravská cesta 9, 84505, Bratislava, Slovakia; Metabiota, UNITED STATES

## Abstract

Tudor staphylococcal nuclease (Tudor-SN) and Argonaute (Ago) are conserved components of the basic RNA interference (RNAi) machinery with a variety of functions including immune response and gene regulation. The RNAi machinery has been characterized in tick vectors of human and animal diseases but information is not available on the role of Tudor-SN in tick RNAi and other cellular processes. Our hypothesis is that tick Tudor-SN is part of the RNAi machinery and may be involved in innate immune response and other cellular processes. To address this hypothesis, *Ixodes scapularis* and *I*. *ricinus* ticks and/or cell lines were used to annotate and characterize the role of Tudor-SN in dsRNA-mediated RNAi, immune response to infection with the rickettsia *Anaplasma phagocytophilum* and the flaviviruses TBEV or LGTV and tick feeding. The results showed that Tudor-SN is conserved in ticks and involved in dsRNA-mediated RNAi and tick feeding but not in defense against infection with the examined viral and rickettsial pathogens. The effect of *Tudor-SN* gene knockdown on tick feeding could be due to down-regulation of genes that are required for protein processing and blood digestion through a mechanism that may involve selective degradation of dsRNAs enriched in G:U pairs that form as a result of adenosine-to-inosine RNA editing. These results demonstrated that Tudor-SN plays a role in tick RNAi pathway and feeding but no strong evidence for a role in innate immune responses to pathogen infection was found.

## Introduction

RNA interference (RNAi) is conserved in eukaryotes with a variety of functions including immune response and gene regulation [1]. Analysis of the RNAi pathways in different model organisms suggests that both Tudor staphylococcal nuclease (Tudor-SN) and Argonaute (Ago) are components of the basic RNAi machinery [2]. The basic RNAi machinery contains a Dicer endonuclease of the RNase III family (Dcr) that processes long double-stranded RNAs (dsRNAs) into small interfering RNAs (siRNAs), and an Ago2 slicer endonuclease that in complex with siRNA and Tudor-SN forms the RNA-induced silencing complex (RISC) that cleaves target mRNA [1]. Ago2 and Dcr2 proteins are essential for the insect antiviral RNAi pathway while Ago1 and Dcr1 proteins are involved in the insect micro RNA (miRNA) pathway [3]. Tudor-SN has been implicated in eukaryotes in a variety of cellular processes such as transcription, processing of dsRNA, RNAi, splicing regulation and stress response [4–6]. However, although RNAi has been evolutionarily conserved in eukaryotes, in some organisms such as *Leishmania* and *Trypanosoma* the RNAi pathways may be inactive through loss of Dicer and/or Ago [7] or some components such as Tudor-SN may play a minor role [8].

The presence of RNAi mechanisms in ticks was first demonstrated by Aljamali et al. [9] and then used as a method for genetic manipulation of ticks [10, 11]. Further evidence of the presence of RNAi pathway genes in ticks was provided by comparative genomics in *Rhipicephalus (Boophilus) microplus* and *Ixodes scapularis* [12, 13]. Additionally, Kurscheid et al. [12] used loss-of-function studies to support a role for putative RNAi pathway genes in *R*. *microplus* development and suggested that these pathways may differ from those present in insects. Aung et al. [14] demonstrated a role for scavenger receptors in dsRNA uptake and systemic RNAi in ticks. RNAi was first implicated in antiviral responses of tick cells by Garcia et al. [15]. Recently, Schnettler et al. [16] demonstrated that *I*. *scapularis* Ago-16 (AgoA) and Ago-30 (AgoD) mediate antiviral activity against tick-borne Langat virus (LGTV) and showed how tick-borne flaviviruses express subgenomic RNAs that interfere with tick RNAi. However, no information is available on the role of Tudor-SN in tick RNAi and other cellular processes.

The *Ixodes* spp. ticks are vectors of bacterial pathogens including *Anaplasma phagocytophilum*, *Borrelia burgdorferi* s.l. and flaviviruses such as tick-borne encephalitis virus (TBEV) that cause disease in humans and animals worldwide [16–19]. Additionally, the *I*. *scapularis* genome is the only assembled tick genome, providing the best resource to annotate putative tick RNAi pathway genes. The *I*. *ricinus* genome has not been sequenced but *de novo* transcriptomics data supports a high degree of sequence identity between *I*. *scapularis* and *I*. *ricinus* [20]. Therefore, these tick species are good candidates for the characterization of tick-pathogen interactions and the role of selected genes at the tick-pathogen interface.

Our hypothesis was that tick Tudor-SN is part of the RNAi machinery and may be involved in innate immune response and other cellular processes. To address this hypothesis, *I*. *scapularis* and *I*. *ricinus* ticks and/or cell lines were used to annotate and characterize the role of Tudor-SN in tick dsRNA-mediated RNAi and other cellular processes. The results showed that Tudor-SN is involved in tick dsRNA-mediated RNAi and feeding but not in defense against infection with the flaviviruses TBEV and LGTV and the rickettsia *A*. *phagocytophilum*.

## Results and Discussion

### The gene encoding putative Tudor-SN is conserved in the *I*. *scapularis* genome

**Fig 1:**
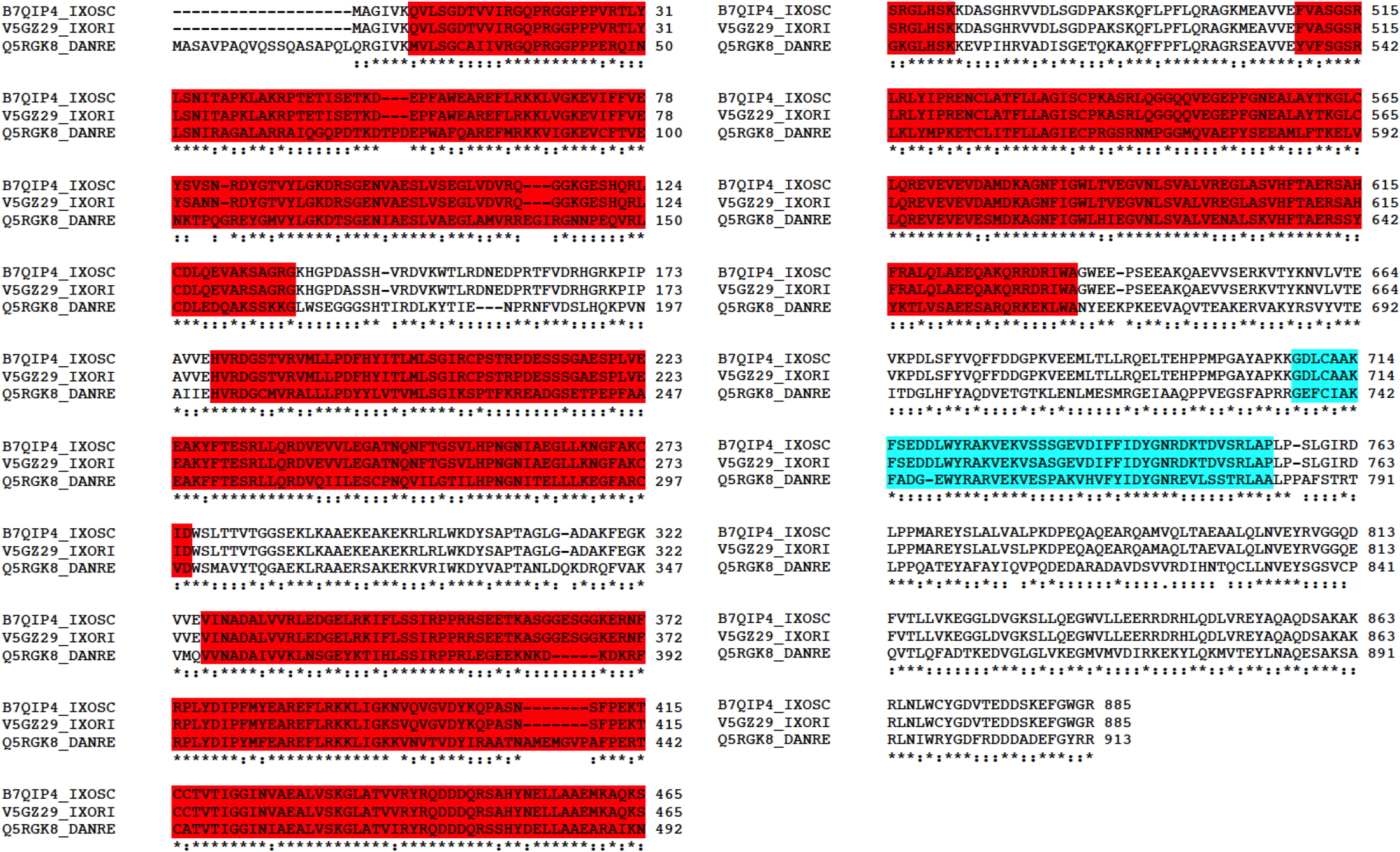
Structure of the *I*. *scapularis* Tudor-SN. Tudor-SN amino acid sequence alignment between *I*. *scapularis* (B7QIP4_IXOSC), *I*. *ricinus* (V5GZ29_IXORI) and *Danio rerio* (Q5RGK8_DANRE). Conserved domains in *I*. *scapularis* Tudor-SN contain conserved staphylococcal nuclease homologues (SN, cd00175; red) and Tudor domain (cd04508; blue).

**Fig 2:**
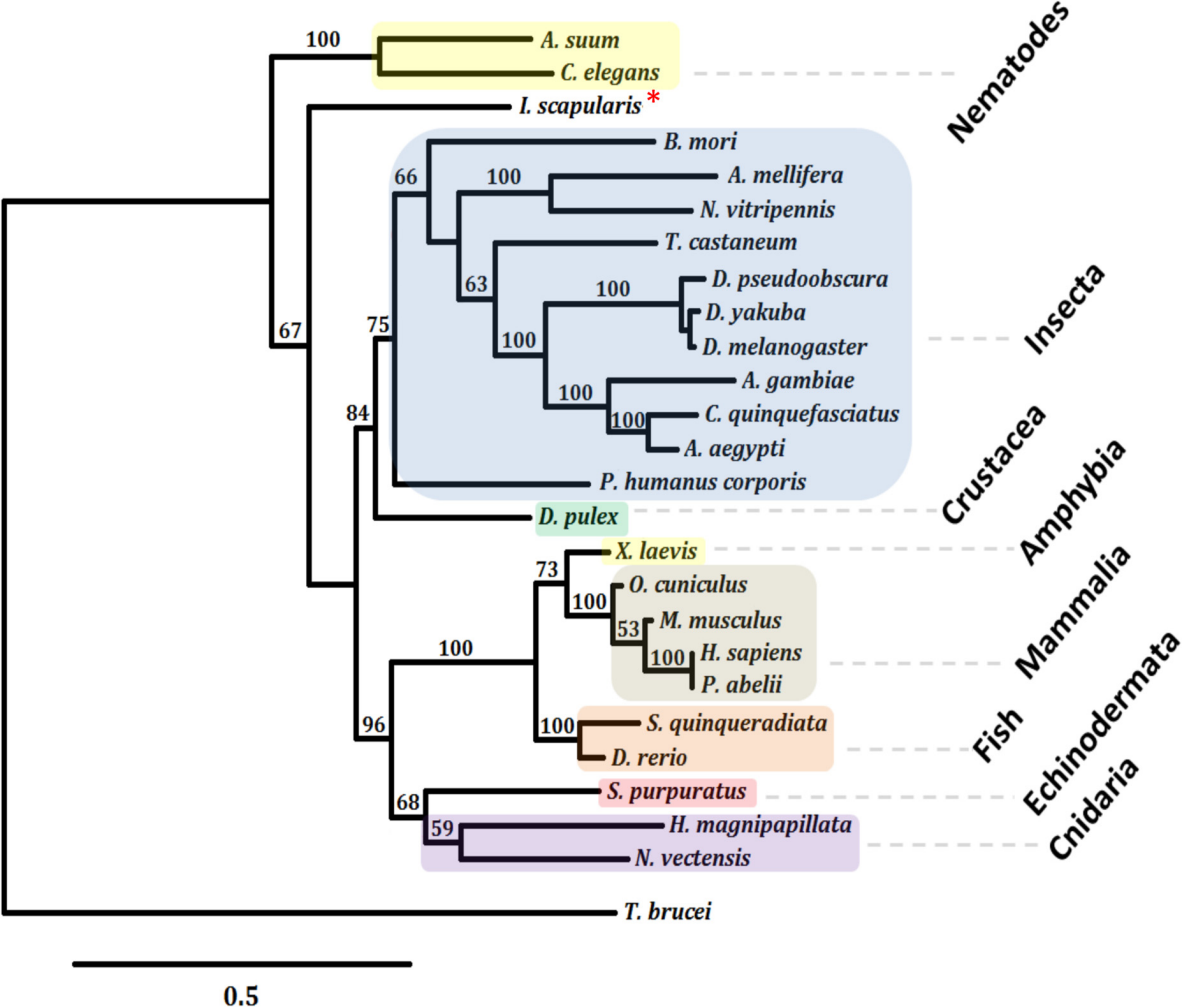
Phylogenetic position of the *I*. *scapularis* Tudor-SN. Amino acid sequences of Tudor-SN orthologs from several taxa (coloured branches) were aligned with *I*. *scapularis* Tudor-SN (red asterisk) using MAFFT. Phylogenetic analyses using ML (shown) and NJ were conducted with similar results. Numbers on internal branches are bootstrapping values. Only bootstrap values higher than 50 are shown.

One putative Tudor-SN encoding gene (ISCW014289) was identified in the *I*. *scapularis* genome. Sequence homology between *I*. *scapularis* and *I*. *ricinus* Tudor-SN sequences was 97% and 99% for nucleotide and amino acid sequences, respectively. The structure of the *I*. *scapularis* Tudor-SN was similar to that described in other species with conserved staphylococcal nuclease homologues (SN) and Tudor domains [21] (Fig 1). The SN and Tudor domains were 99% and 100% identical between *I*. *scapularis* and *I*. *ricinus* protein sequences, respectively (Fig 1). The *I*. *scapularis* Tudor-SN clustered close to reported Insecta sequences (Fig 2).

### *I*. *scapularis* Tudor-SN is involved in dsRNA-mediated RNAi

For the characterization of the putative role of Tudor-SN in dsRNA-mediated RNAi in ticks, an experiment was conducted in *I*. *scapularis* ISE6 tick cells. The experiment was based on using dsRNA targeting *Tudor-SN* (Tudor domain) and Subolesin (*SUB*) to see the effect of *Tudor-SN* gene knockdown on *SUB* expression. A similar experimental approach demonstrated the role of *Panaeus monodon* Tudor-SN in RNAi [22].

The results of the double-knockdown experiments showed that *Tudor-SN* gene knockdown prevented *SUB* knockdown (Table 1). To rule out off-target effects, the experiment was repeated using another dsRNA targeting tick *Tudor-SN* (SN instead of Tudor domain) and the results confirmed previous results using Tudor domain-targeted dsRNA (Table 1). These results demonstrated that Tudor-SN is involved in dsRNA-mediated RNAi in ticks and prompted the question of whether this protein is involved in tick innate immune responses and other cellular processes.

**Table 1:** Role of Tudor-SN in tick dsRNA-mediated RNAi.

Experimental group (injected dsRNA)	*Tudor-SN* / *AgoA* expression silencing (%)	*SUB* expression silencing (%)
*Tudor-SN* + *SUB*	89±13^a^, 91±17^b^	NS^a,b^
*Tudor-SN*	65±36^a^, 62±19^b^	NS^a,b^
*SUB*	NS for *AgoA* and *Tudor-SN* ^a,b^	67±8^a^, 69±5^b^

Ticks were injected with dsRNA targeting *Tudor-SN* (^a^Tudor or ^b^SN domain) and *SUB* to see the effect of *Tudor-SN* gene knockdown on *SUB* expression. Gene knockdown was evaluated with respect to *Rs86* control. Statistical analysis was conducted with normalized Ct values between test and control groups by Students t-test. Only significant values (P<0.05) are shown. Two independent experiments with 20 ticks each were conducted. Values are shown as Ave±SD (number of independent experimental replicates (N) = 20). Abbreviation: NS, not significant.

### *I*. *scapularis* Tudor-SN is not involved in defense against flaviviral or *A*. *phagocytophilum* rickettsial infection

To characterize the possible role of Tudor-SN in the tick innate immune response to viral and rickettsial infection, experiments were conducted using *Ixodes* spp. ticks and/or tick cell lines infected with the rickettsia *A*. *phagocytophilum* and the flaviviruses TBEV or LGTV. *I*. *scapularis* and *I*. *ricinus* were selected because these tick species are the natural vectors for the *A*. *phagocytophilum* and TBEV isolates used in the study, respectively. The LGTV closely related to TBEV but with low pathogenicity and lack of naturally-occurring cases of disease in humans and animals was used to infect both *I*. *scapularis* and *I*. *ricinus* tick cells as a useful experimental model for more virulent tick-borne flavivirus infections [16].

**Fig 3:**
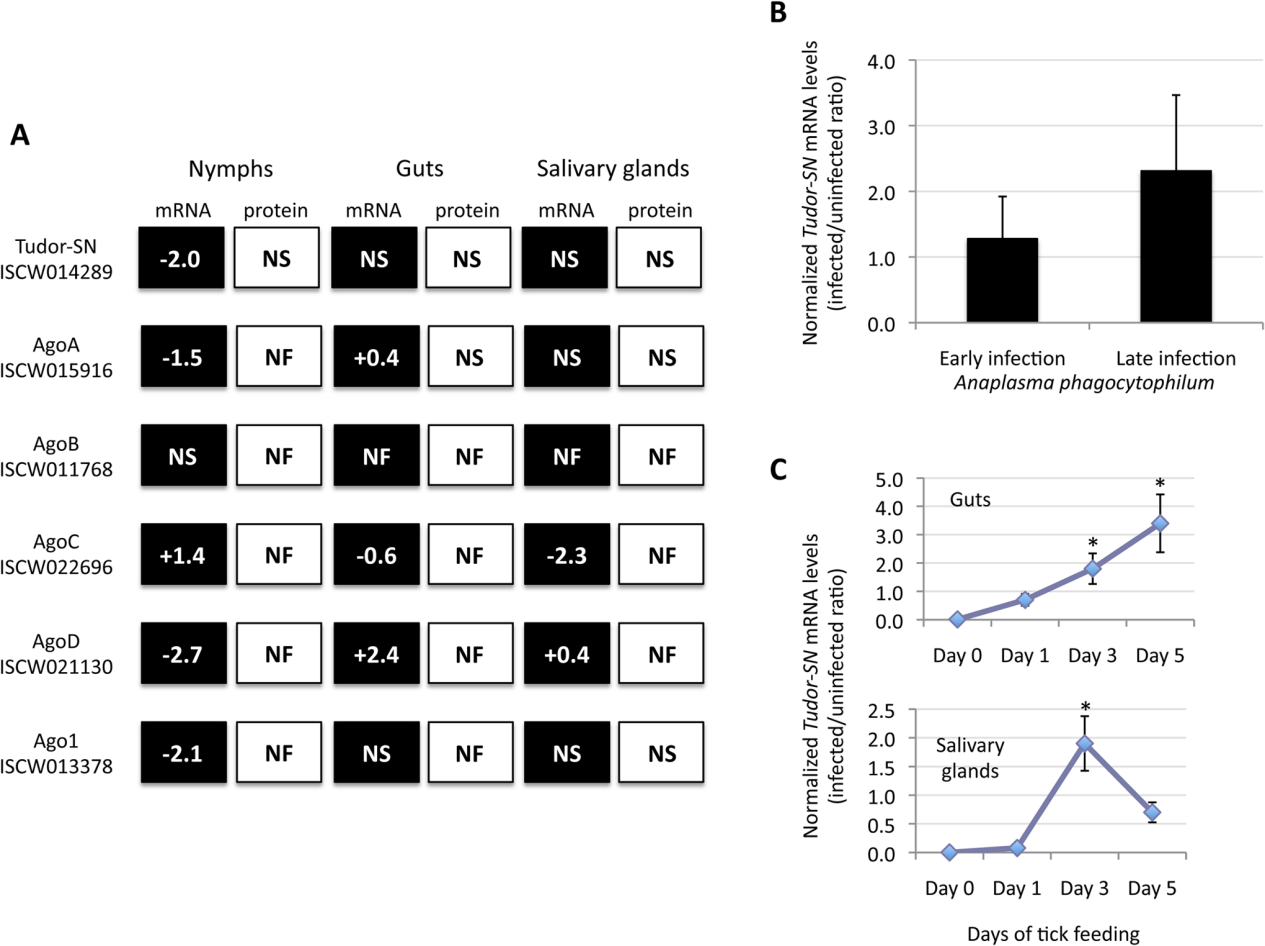
*Tudor-SN* expression in response to pathogen infection. (A) Differential expression/representation of Tudor-SN and Ago genes/proteins in *I*. *scapularis* nymphs, adult female guts and salivary glands in response to infection with *A*. *phagocytophilum*. Data was obtained from transcriptomics and proteomics analyses and values are shown as infected/uninfected Log2-fold ratio (P<0.05). Abbreviations:-, down-regulated/under-represented in infected ticks; +, up-regulated/over-represented in infected ticks; NS, no significant difference between infected and uninfected ticks; NF, not found in trascriptomics or proteomics data. (B) *Tudor-SN* mRNA levels in *I*. *scapularis* ISE6 cells in response to early (day 6) and late (day 13) infections with *A*. *phagocytophilum*. Five independent experiments were conducted for each early and late infection. *Tudor-SN* mRNA levels were determined by real-time RT-PCR in uninfected and infected cells and the infected-to-uninfected ratio of Ct values normalized against tick *16S rRNA* and *cyclophilin* are shown in arbitrary units (Ave+SD). Normalized *Tudor-SN* mRNA levels were compared by Student’s t-test with unequal variance and were not statistically different between infected and uninfected cells (P = 0.05; N = 5). (C) *Tudor-SN* mRNA levels in *I*. *ricinus* ticks during TBEV infection. *Tudor-SN* mRNA levels were determined by real-time RT-PCR in the guts and salivary glands of female ticks (N = 10 ticks for each time point) uninfected and artificially infected with TBEV and fed on mice for 0, 1, 3 and 5 days. *Tudor-SN* Ct values normalized against tick *16S rRNA* are shown in arbitrary units (Ave±SD) and were used to calculate infected/uninfected ratios and compared between Days 1–5 and Day 0 by Student’s t-test with unequal variance (*P<0.05; N = 10).

First, *Tudor-SN* mRNA levels were characterized in response to rickettsial infection. Using transcriptomics and proteomics data generated from *I*. *scapularis* nymphs, adult female guts and salivary glands in response to *A*. *phagocytophilum* infection [23], the results showed that while in *I*. *scapularis* nymphs *Tudor-SN* mRNA levels were down-regulated in response to infection with *A*. *phagocytophilum*, rickettsial infection did not affect *Tudor-SN* expression in guts and salivary glands collected from adult female ticks fed on an infected sheep for 7 days (Fig 3A). Furthermore, Tudor-SN protein levels were not affected in response to *A*. *phagocytophilum* infection in *I*. *scapularis* nymphs and adult tissues (Fig 3A). The differences between mRNA and protein levels could be due, at least in part, by delay between mRNA synthesis and protein accumulation and/or the role for post-transcriptional and post-translational modifications in tick cell response to *A*. *phagocytophilum* infection [23]. The pattern of mRNA and protein levels for the five Ago paralogs described in *I*. *scapularis* [16] differed from those observed for Tudor-SN, providing additional evidence for the different role of these molecules in response to pathogen infection in ticks (Fig 3A). The fact that particular transcripts or proteins were not found in transcriptomics or proteomics data could be due to several factors. It could be that these transcripts or proteins are difficult to detect due to technical problems associated with current technologies and databases. However, if a particular transcript or protein is found in one of the samples but not in others, then it is very likely that mRNA or protein levels were too low to be detected in these samples, which is equivalent to showing no significant differences between infected and uninfected ticks. In ISE6 tick cells, *A*. *phagocytophilum* infection did not affect *Tudor-SN* mRNA levels (Fig 3B). In contrast, TBEV infection resulted in the up-regulation of *Tudor-SN* expression in *I*. *ricinus* adult female guts and salivary glands as feeding progressed (Fig 3C).

**Fig 4:**
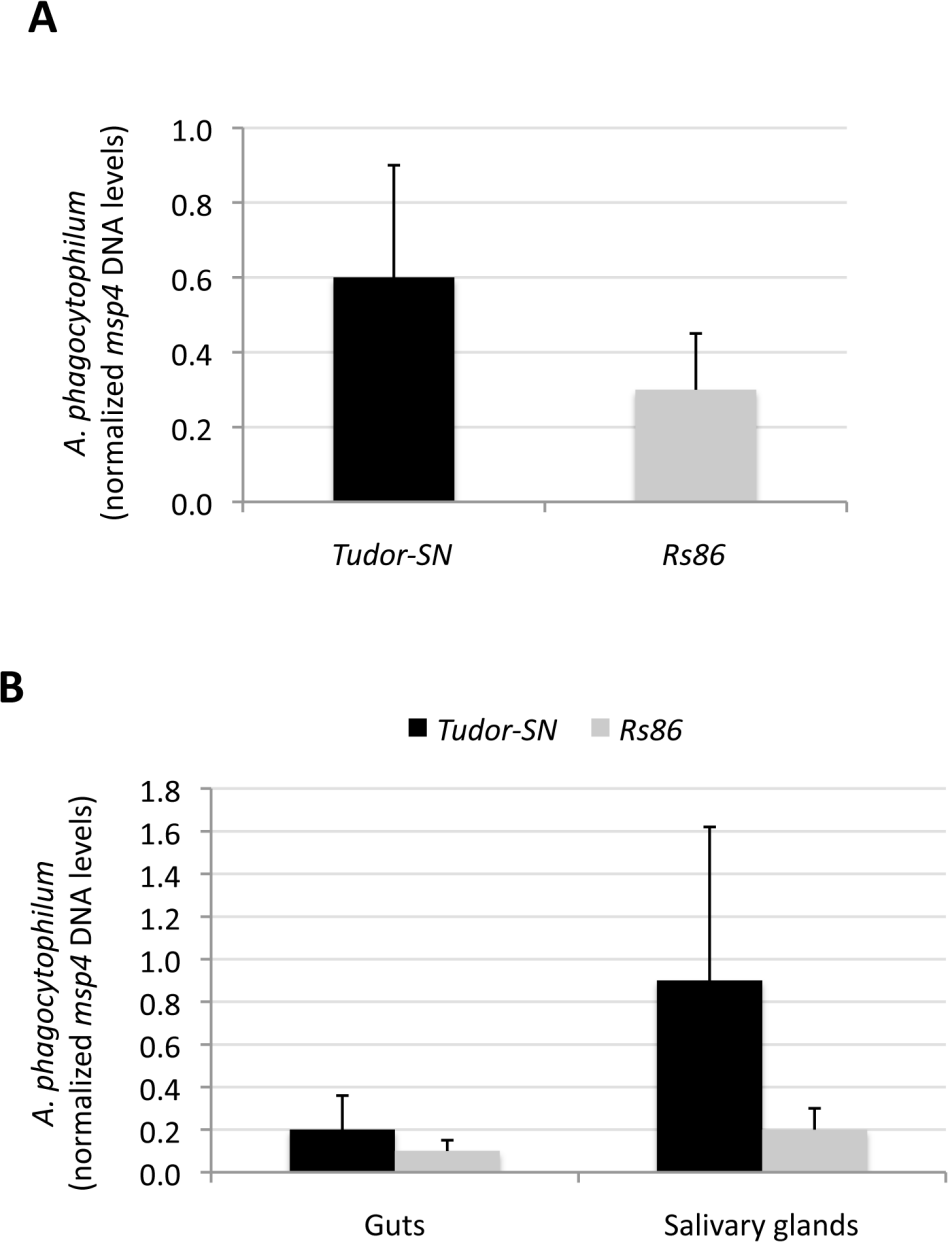
Effect of *Tudor-SN* knockdown on *A*. *phagocytophilum* infection. (A) *I*. *scapularis* ISE6 cells were treated with *Tudor-SN* or *Rs86* control dsRNAs and infected with *A*. *phagocytophilum* NY18. *A*. *phagocytophilum* DNA levels were characterized by *msp4* real-time PCR normalizing against tick *16S rDNA*. Normalized Ct values were compared between groups by Student's t-test with unequal variance and were not statistically different between *Tudor-SN* and *Rs86* dsRNA-treated cells (P = 0.05; N = 4 wells per treatment). (B) *I*. *scapularis* female ticks were injected with *Tudor-SN* or *Rs86* control dsRNAs and infected with *A*. *phagocytophilum* NY18 by feeding on an infected sheep. *A*. *phagocytophilum* DNA levels were characterized in tick guts and salivary glands by *msp4* real-time PCR normalizing against tick *16S rDNA*. Normalized Ct values were compared between groups by Student's t-test with unequal variance and were not statistically different between *Tudor-SN* and *Rs86* dsRNA-injected ticks (P = 0.05; N = 10 ticks per group).

The RNAi pathway has not been reported to be involved in tick response to rickettsial infection but RNAi inhibits flavivirus replication in vertebrate [24] and tick [16] cells. The possible role of Tudor-SN in tick innate immune responses was characterized by analyzing the effect of *Tudor-SN* gene knockdown on *A*. *phagocytophilum* and LGTV replication in infected tick cells. *Tudor-SN* gene knockdown in ISE6 tick cells (81±11% silencing; N = 4, P<0.01) and adult female guts (50±4% silencing; N = 10, P<0.01) and salivary glands (94±5% silencing; N = 10, P<0.01) did not affect *A*. *phagocytophilum* infection when compared to the *Rs86* dsRNA control (Fig 4A and 4B).

**Fig 5:**
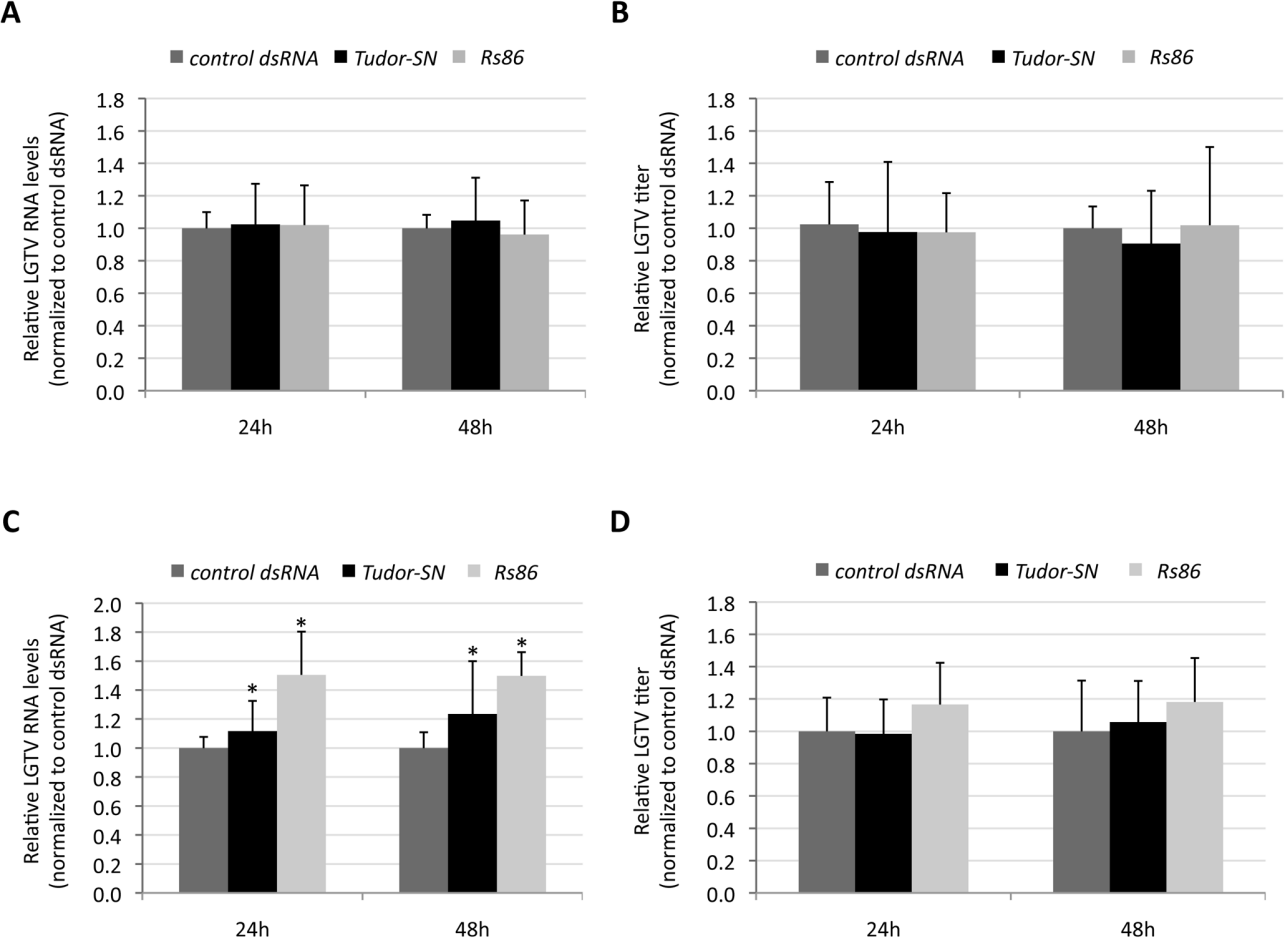
Effect of *Tudor-SN* knockdown on LGTV infection. (A) *I*. *scapularis* IDE8 cells were treated with *Tudor-SN*, *Rs86* or *GFP* control dsRNAs and infected with LGTV at MOI 0.1. LGTV RNA levels were determined after 24h and 48h by *NS5* real-time PCR normalizing against tick *β-actin* mRNA levels and are presented relative to the *GFP* dsRNA control. Relative LGTV RNA levels were compared between groups by two-way ANOVA and were not statistically different between *Tudor-SN* and *GFP* dsRNA-treated cells (P>0.05; N = 8). (B) *I*. *scapularis* IDE8 cells were treated with *Tudor-SN*, *Rs86* or *GFP* control dsRNAs and infected with LGTV at MOI 0.1. Virus titers in the cell supernatants were determined by plaque assay after 24h and 48h and are presented relative to the *GFP* dsRNA control. Relative LGTV titers were compared between groups by two-way ANOVA and were not statistically different between *Tudor-SN* and *GFP* dsRNA-treated cells (P>0.05; N = 8). (C) *I*. *ricinus* IRE/CTVM20 cells were treated with *Tudor-SN*, *Rs86* or *GFP* control dsRNAs and infected with LGTV at MOI 0.1. LGTV RNA levels were determined after 24h and 48h by *NS5* real-time PCR normalizing against tick *β-actin* mRNA levels and are presented relative to the *GFP* dsRNA control. Relative LGTV RNA levels were compared between groups by two-way ANOVA (P≤0.05; N = 8). (D) *I*. *ricinus* IRE/CTVM20 cells were treated with *Tudor-SN*, *Rs86* or *GFP* control dsRNAs and infected with LGTV at MOI 0.1. Virus titers in the cell supernatants were determined by plaque assay after 24h and 48h and are presented relative to the *GFP* dsRNA control. Relative LGTV titers were compared between groups by two-way ANOVA and were not statistically different between *Tudor-SN* and *GFP* dsRNA-treated cells (P>0.05; N = 8). For each tick cell line, two independent experiments with 4 replicates each were conducted and combined after normalization.

In IDE8 and IRE/CTVM20 tick cells, *Tudor-SN* gene knockdown (85±2% and 90±3% and 38±1% and 42±2% silencing at 24 and 48 hours post-infection in IDE8 and IRE/CTVM20 tick cells, respectively; N = 8, P<0.01) did not affect LGTV replication when compared to *Rs86* and *GFP* dsRNA controls (Fig 5A–5D). The effect of gene knockdown on relative LGTV RNA levels in IRE/CTVM20 cells (Fig 5C) was similar for both *Tudor-SN* dsRNA and *Rs86* control dsRNA and was therefore not specific for Tudor-SN.

These results suggested that Tudor-SN is not involved in tick innate immune responses to infection with *A*. *phagocytophilum* and LGTV. However, as demonstrated by Schnettler et al. [16], it is possible that flavivirus-expressed subgenomic RNAs interfere with the tick RNAi mechanism and thus masked the role of Tudor-SN in this process.

### *I*. *scapularis* Tudor-SN is involved in tick feeding

Although the experiments reported here did not indicate a role for Tudor-SN in the tick innate immunity to infection with flaviviruses or *A*. *phagocytophilum*, it could be involved in other cellular processes such as transcription, processing of dsRNA, splicing regulation and stress response [4–6]. In ticks, blood feeding is an essential process that requires the function of several proteins including proteases and protease inhibitors [25, 26], some of which could be regulated by Tudor-SN as reported in other organisms [5].

**Fig 6:**
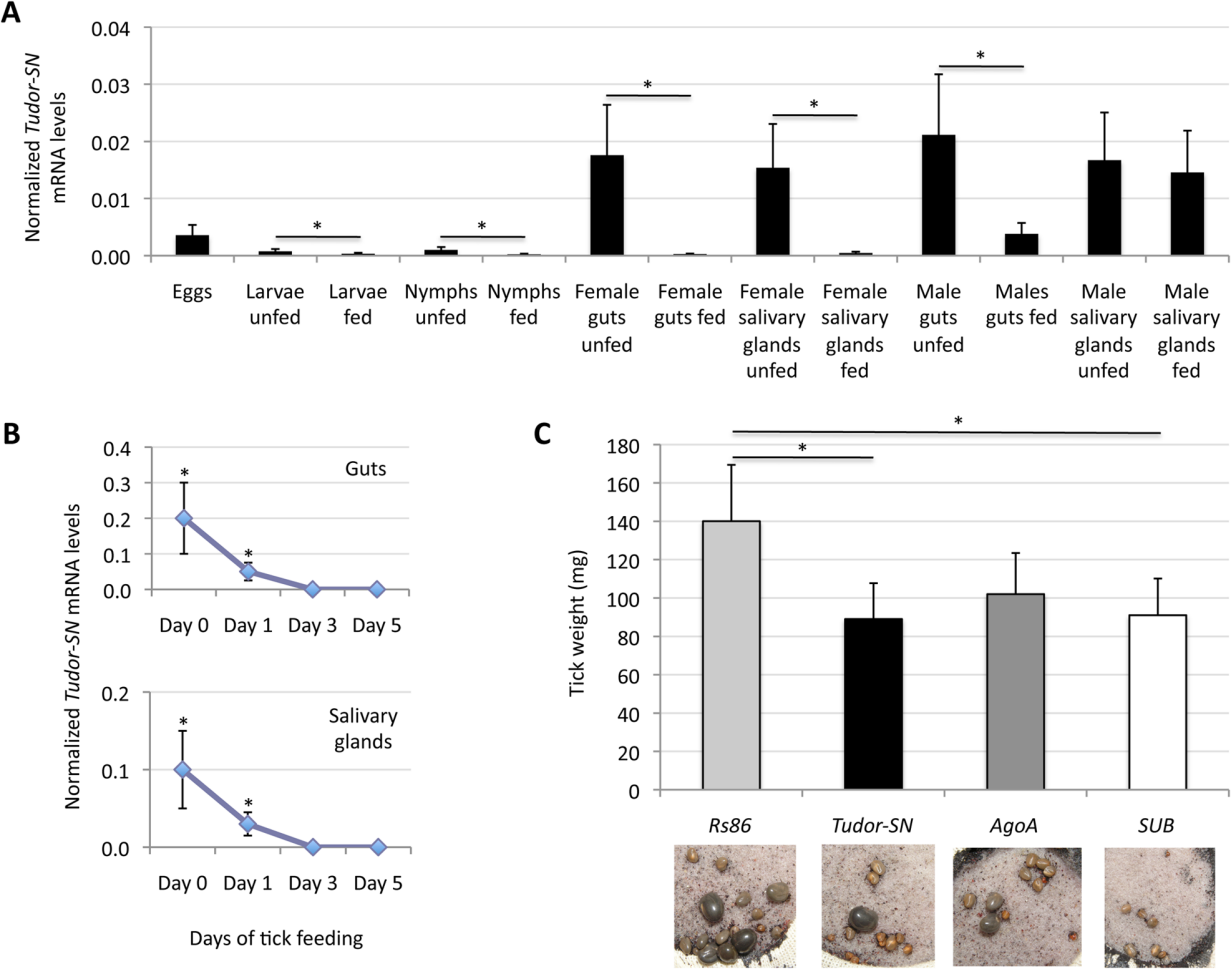
Effect of *Tudor-SN* knockdown on tick feeding. (A) *Tudor-SN* mRNA levels were analyzed using RNA extracted from *I*. *scapularis* eggs (three batches of approximately 500 eggs each), fed and unfed larvae (three pools of 50 larvae each), fed and unfed nymphs (three pools of 15 nymphs each), and fed and unfed male and female adult tick tissues (4 ticks each) by real-time RT-PCR and normalizing against tick *cyclophilin* and *ribosomal protein S4*. Normalized Ct values were compared between unfed and fed ticks by Student's t-test with unequal variance (*P<0.05). (B) *Tudor-SN* mRNA levels in *I*. *ricinus* ticks during tick feeding. *Tudor-SN* mRNA levels were determined by real-time RT-PCR in the guts and salivary glands of uninfected female ticks (N = 10 ticks for each time point) fed on mice for 0 (unfed ticks), 1, 3 and 5 days. *Tudor-SN* Ct values normalized against tick *16S rRNA* are shown in arbitrary units (Ave±SD) and were compared between Days 0–3 and Day 5 by Student's t-test with unequal variance (*P<0.05). (C) *I*. *scapularis* female ticks were injected with dsRNA and fed on sheep. Tick weights (Ave+SD) were compared between groups by Student's t-test with unequal variance (*P<0.05; N = 20 ticks per group). Panels show representative images at day 5 of tick feeding.

To test this hypothesis, the effect on tick feeding of *Tudor-SN* gene knockdown was characterized. Apart from in male salivary glands, *Tudor-SN* mRNA levels were always significantly lower in fed than unfed tick samples (Fig 6A) and decreased during female tick feeding in both guts and salivary glands (Fig 6B), suggesting a role for this molecule during feeding. The effect of tick feeding on decreasing *Tudor-SN* mRNA levels was counteracted by TBEV infection in both guts and salivary glands (Fig 3C). Furthermore, *Tudor-SN* gene knockdown (Table 1) resulted in 36% reduction of tick weight similar to that seen with *SUB* [27] in fed *I*. *scapularis* females when compared to ticks injected with *AgoA* and *Rs86* control dsRNA (Fig 6C).

**Fig 7:**
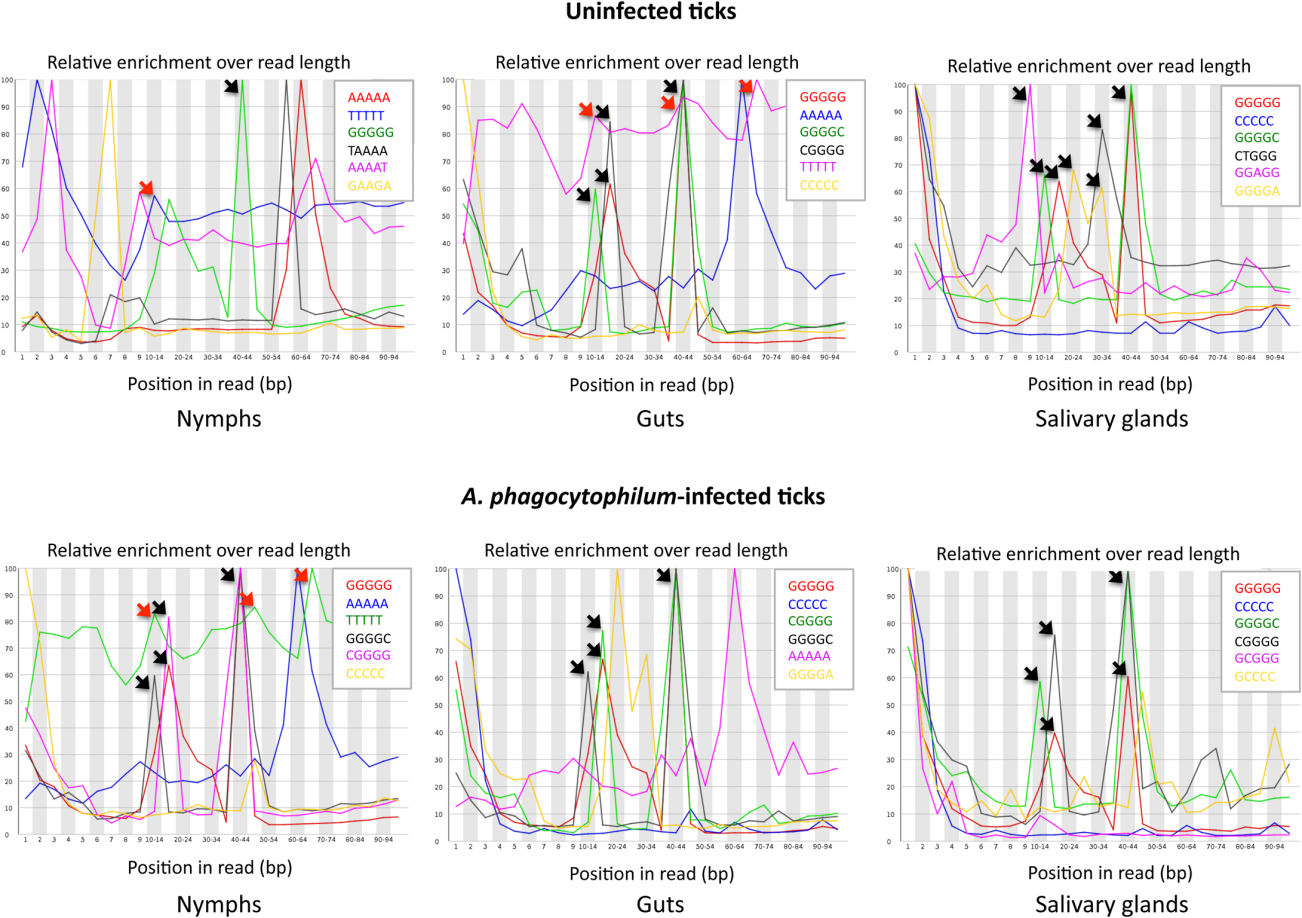
Overrepresented k-mers in *I*. *scapularis* tick transcriptome. Profile of k-mers in transcriptomics data generated from uninfected and *A*. *phagocytophilum*-infected *I*. *scapularis* nymphs and adult female guts and salivary glands. Overrepresented T-rich (red arrows) and/or G-rich (black arrows) sequences were identified in the central part (nucleotides 10–70 of 100) of all samples. The overall %GC content for the transcriptome in all samples was 48±6. Data shown represents results of two replicates. Produced by FastQC (version 0.9.1; http://www.bioinformatics.bbsrc.ac.uk/projects/fastqc/).

The effect of *Tudor-SN* gene knockdown on tick feeding could be due to down-regulation of genes that are required for protein processing and blood digestion [5]. The mechanism by which Tudor-SN regulates gene expression in ticks is unknown, but as shown in animal cells it could act as a global regulator of gene expression through selective degradation of dsRNAs enriched in G:U pairs that form as a result of adenosine-to-inosine RNA editing [28]. Inosine base pairs with cytidine during reverse transcription and therefore appears as G during cDNA sequencing [29]. One of the approaches for finding possible targets for RNA-binding proteins is the use of identified overrepresented k-mers in a set of sequences [30]. Using transcriptomics data generated from uninfected and *A*. *phagocytophilum*-infected *I*. *scapularis* nymphs and adult female guts and salivary glands [23], a set of overrepresented k-mers was identified (Fig 7). Apart from sequencing errors that could explain some of these k-mers particularly at both ends of the sequence, the results showed that overrepresented T-rich and/or G-rich sequences were identified in the central part (nucleotides 10–70 of 100) of all samples, suggesting the possibility of G:U pairs to explain the relatively low alignment to the reference genome obtained for some reads [23]. Particularly relevant was the presence of both T-rich and G-rich sequences in nymphs and adult female guts, where blood digestion takes place. Interestingly, the number of G-rich and T-rich regions increased with *A*. *phagocytophilum* infection only in nymphs (Fig 7), which was the only sample in which *Tudor-SN* expression was down-regulated in response to infection (Fig 3A). These results suggested that enriched G:U sequences could form in ticks and be targeted by Tudor-SN. Consequently, *Tudor-SN* gene knockdown may affect the expression of genes encoding proteins involved in protein processing and blood digestion, thus affecting tick feeding. However, further experiments are required to test this hypothesis.

## Conclusions

The results of this study showed that Tudor-SN is a conserved component of the basic RNAi machinery in *Ixodes* ticks. Tick Tudor-SN had an effect on dsRNA-mediated gene silencing and therefore possibly the siRNA pathway. No strong evidence was obtained for a role of Tudor-SN in tick innate immune response to pathogen infection, suggesting that pathogens may have developed mechanisms to subvert this protective mechanism in ticks [16] while other pathways are activated to control infection [23]. However, Tudor-SN was found to be involved in tick feeding possibly through down-regulation of genes that are required for protein processing and blood digestion through a mechanism that may involve selective degradation of dsRNAs enriched in G:U pairs that form as a result of adenosine-to-inosine RNA editing. It has been reported that *A*. *phagocytophilum* reduces the expression of certain tick genes such as mitochondrial *porin* to facilitate infection without affecting tick feeding [23]. In the present study, considering the effect of *Tudor-SN* gene knockdown on tick weight and that in most cases Tudor-SN levels did not change or increase after pathogen infection suggested that pathogens subverted tick RNAi response by mechanisms other than reducing Tudor-SN levels to preserve tick feeding and thus vector capacity. These results highlighted co-evolutionary mechanisms by which pathogens manipulate tick immune response to facilitate infection but preserving tick feeding and vector capacity to guarantee survival of both pathogens and ticks.

## Materials and Methods

### Annotation of putative Tudor-SN in *I*. *scapularis*

For annotation of putative Tudor-SN, *I*. *scapularis* genome sequence assembly JCVI_ISG_i3_1.0—NCBI reference sequence NZ_ABJB000000000 (GenBank accession ABJB010000000; http://www.ncbi.nlm.nih.gov/nuccore/NZ_ABJB000000000) was used. Gene identifiers were obtained from VectorBase (www.vectorbase.org) and compared to the corresponding pathways in *Drosophila melanogaster*, *Anopheles gambiae*, *Aedes aegypti* and *Homo sapiens*. The Conserved Domain Database (http://www.ncbi.nlm.nih.gov/cdd) was used for the annotation of functional units in *I*. *scapularis* Tudor-SN. Multiple sequence alignment was conducted using ClustalW2 (http://www.ebi.ac.uk/Tools/msa/clustalw2/) and BLAST (bl2seq; http://blast.ncbi.nlm.nih.gov) for amino acid and nucleotide sequences, respectively.

### Phylogenetic analysis

The evolutionary history of Tudor-SN protein sequences was inferred using sequences aligned with MAFFT (v7) configured for the highest accuracy [31]. After alignment, regions with gaps were removed from the alignment. Phylogenetic trees were constructed using maximum likelihood (ML) and neighbor joining (NJ) methods as implemented in PhyML (v3.0 aLRT) [32, 33] and PHYLIP (v3.66) [34], respectively. The reliability for the internal branches of ML was assessed using the bootstrapping method (1000 bootstrap replicates) and the approximate likelihood ratio test (aLRT–SH-Like) [33]. Reliability for the NJ tree was assessed using bootstrapping method (1000 bootstrap replicates). Graphical representation and editing of the phylogenetic trees were performed with TreeDyn (v 198.3) [35]. The following organisms and sequences available at GenBank were used in the Tudor-SN phylogenetic analysis: *I*. *scapularis* (XP_002415051); *Daphnia pulex* (EFX77404); *Nematostella vectensis* (XP_001635744); *Strongylocentrotus purpuratus* (XP_798852); *D*. *rerio* (NP_878285); *Pongo abelii* (NP_001125262); *H*. *sapiens* (BAD92747); *Oryctolagus cuniculus* (XP_002712124); *Mus musculus* (NP_062750); *A*. *aegypti* (XP_001654799); *Xenopus laevis* (NP_001079606); *Hydra magnipapillata* (XP_002163890); *Culex quinquefasciatus* (XP_001848537); *Tribolium castaneum* (XP_974879); *Nasonia vitripennis* (NP_001153329); *D*. *melanogaster* (NP_612021); *Seriola quinqueradiata* (BAC65164); *D*. *yakuba* (XP_002092984); *Bombyx mori* (NP_001182009); *Caenorhabditis elegans* (NP_494839); *D*. *pseudoobscura* (XP_001352493); *Apis mellifera* (XP_624638); *Ascaris suum* (ERG82182); *Pediculus humanus corporis* (XP_002422905); *A*. *gambiae* (XP_315689); *Trypanosoma brucei* (XP_829475).

### Tick and mammalian cell lines and pathogens

The ISE6 and IDE8 tick cell lines, derived originally from *I*. *scapularis* embryos (provided by U.G. Munderloh, University of Minnesota, USA) were cultured in L15B medium as described previously [36]. The *I*. *ricinus* embryo-derived cell line IRE/CTVM20 (provided by the Tick Cell Biobank; [37]) was maintained in a 1:1 mixture of L-15 (Leibovitz) and L-15B medium [38]. For these experiments both cell lines were kept at 31°C in a humid environment. Baby hamster kidney (BHK-21) cells were grown in Glasgow Minimum Essential Medium (GMEM) supplemented with 5% newborn calf serum (NBCS), 10% TPB, 100 U/ml penicillin and 100 μg/ml streptomycin. Cells were maintained at 37°C in a humidified atmosphere of 5% CO_2_ in air.

The ISE6 cells were inoculated with the NY18 isolate of *A*. *phagocytophilum* propagated in HL-60 cells and maintained according to the procedures described by de la Fuente et al. [39]. Uninfected cells were cultured in the same way, except with the addition of 1 ml of culture medium instead of infected cells. Uninfected and infected cultures (five independent cultures with approximately 10^7^ cells each) were sampled at early infection (11–17% infected cells (Ave±SD, 13±2)) and late infection (56–61% infected cells (Ave±SD, 58±2)). *A*. *phagocytophilum* infection was determined by *msp4* real-time PCR. LGTV strain TP21, kindly provided by Sonja Best (NIH-National Institute of Allergy and Infectious Diseases, Rocky Mountain Laboratories, Hamilton, MT, USA), was used for infection of IDE8 and IRE/CTVM20 tick cells as described previously [16]. Collected cells were centrifuged at 10,000 x g for 3 min and cell pellets were frozen in liquid nitrogen until used for DNA and RNA extraction.

### Ticks and pathogens

*I*. *scapularis* ticks were obtained from the laboratory colony maintained at the Oklahoma State University Tick Rearing Facility. Larvae and nymphs were fed on rabbits and adults were fed on sheep. Off-host ticks were maintained in a 12 hr light: 12 hr dark photoperiod at 22–25°C and 95% relative humidity. Nymphs and adult female *I*. *scapularis* were infected with *A*. *phagocytophilum* by feeding on a sheep inoculated intravenously with approximately 1x10^7^ *A*. *phagocytophilum* (NY18 isolate)-infected HL-60 cells (90–100% infected cells) [40]. In this model, over 85% of ticks become infected with *A*. *phagocytophilum* in nymphs, guts and salivary glands [40]. Uninfected ticks were prepared in a similar way but feeding on an uninfected sheep. Ten individual nymphs and female ticks were dissected and whole tissues (nymphs), guts and salivary glands (adults) collected to characterize *A*. *phagocytophilum* infection and the mRNA levels of selected genes. Animals were housed and experiments conducted with the approval and supervision of the OSU Institutional Animal Care and Use Committee (Animal Care and Use Protocol, ACUP No. VM1026).

*I*. *ricinus* ticks were obtained from a pathogen-free laboratory colony maintained at the Institute of Zoology (Bratislava, Slovakia; permit number 1335/12-221). Unfed *I*. *ricinus* females were inoculated with TBEV, Hypr strain (Institute of Virology SAS, Bratislava; 3x10^4^ PFU per tick) under a stereo zoom microscope (Wild M 400, Wild Heerbrugg AG, Switzerland) into the coxal plate of the second pair of legs using a digital microinjector TM system (MINJ-D-CE; Tritech Research, Inc., USA) [41]. Clean nitrogen served as a gas source to produce an injection pressure of 20 psi (approx. 1.38 bar). The injection interval was set to 1.0 sec. Hollow glass needles with a microscopically fine tip were prepared using a P-30 Micropipette puller (Sutter Instrument Company, USA). Injected ticks were incubated at room temperature and 85–90% relative humidity in a desiccator for 21 days prior to the experiments. Adult female ticks (N = 10 for each time point) were inoculated with TBEV and fed on 6-week-old pathogen-free BALB/c mice (Masaryk University in Brno, Czech Republic) for 0 (unfed ticks), 1, 3, and 5 days and removed for dissection of adult female guts and salivary glands. All animal experiments were approved by the State Veterinary and Food Administration of the Slovak Republic (permit number 2976/09-221). None of the animals in which ticks were fed became ill.

### Transcriptomics and proteomics analyses

Transcriptomics and proteomics analyses were conducted using duplicate RNA and protein samples extracted from uninfected and *A*. *phagocytophilum*-infected *I*. *scapularis* nymphs and adult female guts and salivary glands as previously described [23]. Briefly, RNA sequencing was done using 100 bp paired-end reads on Illumina Hiseq 2000 (CD BioSciences, Shirley, NY, USA). TopHat [42] was used to align the reads to the *I*. *scapularis* (assembly JCVI_ISG_i3_1.0; http://www.ncbi.nlm.nih.gov/nuccore/NZ_ABJB000000000) reference genome. Raw counts per gene were estimated by the Python script HTSeq count (http://www-huber.embl.de/users/anders/HTSeq/) using the reference genome. The raw counts per gene were used by DEGseq [43] to estimate differential expression at P<0.05. The k-mers analysis was performed with FastQC (version 0.9.1; http://www.bioinformatics.bbsrc.ac.uk/projects/fastqc/).

Proteins were digested using the filter aided sample preparation (FASP) protocol [44]. For stable isobaric labeling, the resulting tryptic peptides were dissolved in Triethylammonium bicarbonate (TEAB) buffer and labeled using the 4-plex iTRAQ Reagents Multiplex Kit (Applied Biosystems, Foster City, CA, USA) according to manufacturer's protocol. Labeled peptides were loaded into the LC-MS/MS system for on-line desalting onto C18 cartridges and analyzing by LC-MS/MS using a C-18 reversed phase nano-column (75 μm I.D. x 50 cm, 3 μm particle size, Acclaim PepMap 100 C18; Thermo Fisher Scientific, Waltham, MA, USA) [23]. For peptide identification, all spectra were analyzed with Proteome Discoverer (version 1.4.0.29, Thermo Fisher Scientific) using a Uniprot database containing all sequences from Ixodida (May 17, 2013). Peptide identification was validated using the probability ratio method [45] and false discovery rate (FDR) was calculated using inverted databases and the refined method [46] with an additional filtering for precursor mass tolerance of 12 ppm. Only peptides with a confidence of at least 95% were used to quantify the relative abundance of each peptide determined as described previously [47]. Protein quantification from reporter ion intensities and statistical analysis of quantitative data were performed as described previously using QuiXoT [48, 49]. For iTRAQ data, only the intensity of the reporter ions within 0.4 Da windows around the theoretical values was considered for quantification. Reporter intensities were corrected for isotopic contaminants by taking into consideration the information provided by the manufacturer. The intensity of the reporter peaks was used to calculate the fitting weight of each spectrum in the statistical model as described previously [49]. Outliers at the scan and peptide levels and significant protein-abundance changes were detected from the z values (the standardized variable used by the model that expresses the quantitative values in units of standard deviation) by using a false discovery rate (FDR) threshold of 5% as described previously [49]. Results were the mean of two replicates. All relevant data including accession to transcriptomics and proteomics mediated data can be found in Ayllón et al. [23].

### Gene knockdown by RNAi in ticks

For RNAi, oligonucleotide primers containing T7 promoter sequences (underlined) were synthesized for *I*. *scapularis Tudor-SN* (XP_002415051; TUDT75: 5’-TAATACGACTCACTATAGGGTACTTGGAGGAGATGCTGACACTG-3’ and TUDT73: 5’-TAATACGACTCACTATAGGGTACTTCCTTCGAGTCGTCCTCTGT-3’ targeting Tudor domain or SNT75: 5‘-TAATACGACTCACTATAGGGTACTTGTGGAGTACAGCGTAAGC-3’ and TUDT73: 5’-TAATACGACTCACTATAGGGTACTTCAATGCACTTGGCAAAGCC-3’ targeting SN domain) and *AgoA* (XM_002401346; Ago1T75: 5’-TAATACGACTCACTATAGGGTACTAAGGGTGATCAGAAGCTCCA-3’ and Ago1T73: 5’-TAATACGACTCACTATAGGGTACTGTTCCGACCAAGGACGACTA-3’) and used for *in vitro* transcription and synthesis of dsRNA as described previously [27], using the Access RT-PCR system (Promega) and the Megascript RNAi kit (Ambion, Austin, TX, USA). The unrelated gene *Rs86* dsRNA and *SUB* dsRNA were synthesized using the same methods described previously and used as negative and positive controls, respectively [27]. The dsRNA was purified and quantified by spectrophotometry. Unfed *I*. *scapularis* adult female ticks (N = 20 per group) were injected with approximately 0.5 μl dsRNA (5x10^10^-5x10^11^ molecules/μl) in the lower right quadrant of the ventral surface of the exoskeleton [50]. The injections were done using a 10-μl syringe with a 1-inch, 33 gauge needle (Hamilton, Bonaduz, Switzerland). Control ticks were injected with the unrelated Rs86 dsRNA or were left uninjected. After dsRNA injection, female ticks were held in a humidity chamber for 1 day after which they were allowed to feed on an uninfected sheep or on a sheep inoculated intravenously with *A*. *phagocytophilum* (NY18 isolate) as described above with 20 male ticks per tick feeding cell [40]. Unattached female ticks were removed 48 hours after infestation. Female ticks were collected at repletion, weighed and dissected for DNA and RNA extraction using Tri Reagent (Sigma-Aldrich, St. Louis, MO, USA) following manufacturer instructions. RNA was used to characterize gene knockdown by real-time RT-PCR with respect to Rs86 control and DNA was used to characterize *A*. *phagocytophilum* infection by PCR [27]. Tick weight was compared between ticks injected with test genes dsRNA and Rs86 control dsRNA by Student's t-test with unequal variance (P = 0.05).

### Gene knockdown by RNAi in tick cells

RNAi was used to characterize the effect of *Tudor-SN* gene knockdown on tick cell pathogen infection and gene expression. ISE6 tick cells were incubated for 48 h with 1 ml growth medium containing 10 μl *Tudor-SN* dsRNA (5 x 10^10^–5 x 10^11^ molecules/μl) prepared as described previously in 24-well plates using 4 wells per treatment (1 x 10^6^ cells/well). Control cells were incubated with the unrelated *Rs86* dsRNA. Cells were inoculated with the NY18 isolate of *A*. *phagocytophilum* as described above. After the incubation, the medium containing dsRNA was removed and replaced with fresh medium and the cells were then incubated for a further 24 h at 31°C, harvested and used for DNA and RNA extraction to characterize pathogen infection by real-time PCR and gene knockdown by real-time RT-PCR with respect to the Rs86 control.

The effect of *Tudor-SN* gene knockdown on LGTV infection was characterized by RNAi in *I*. *scapularis* IDE8 and *I*. *ricinus* IRE/CTVM20 cells. IDE8 and IRE/CTVM20 cells were incubated for 48 h with 1 ml growth medium containing 10 μl *Tudor-SN* dsRNA (5 x 10^10^–5 x 10^11^ molecules/μl), prepared as described previously, in 24-well plates using 4 wells per treatment (1 x 10^6^ cells/well). Control cells were incubated with dsRNA targeting the unrelated genes *Rs86* or green fluorescent protein (*GFP*), which is not expressed in tick cells and was used as a non-specific control. Forty eight h after addition of dsRNA, 50 μl of LGTV stock, previously titrated in BHK21 cells, were added to each test well to give a multiplicity of infection (MOI) of 0.1, and cells were incubated at 31°C. Medium was changed 2 h after addition of virus. Titers of newly-produced LGTV in the cell supernatant were measured 24 and 48 h post-infection by plaque assay in BHK-21 cells. For plaque assay, 10-fold serial dilutions of LGTV-infected samples were prepared using complete GMEM. BHK-21 cells were inoculated with the virus dilutions for 1 h at 37°C before a semi-solid overlay was added to the cells, so that the virus could only infect neighboring cells, which eventually resulted in visible plaques of dead cells. The overlay consisted of one part 2xMEM (Life Technologies 21935–028, Paisley, UK) and one part 1.2% (w/v) Avicel suspension (FMC biopolymer, Avicel RC-591 NF, Philadelphia, USA). BHK-21 cells were fixed 4 days post-infection with 10% neutral-buffered formaldehyde and stained with 0.1% (w/v) toluidine blue to inactivate the virus and visualize the plaques which appeared clear compared to the blue cell monolayer. Plaques were counted in a dilution with between 10 and 100 plaques and the virus titer was determined by multiplying the mean number of plaques by the dilution factor and dividing it by the volume of inoculum (0.4 ml). Virus titers were then normalized to the *GFP* dsRNA control by dividing the titer of each sample by the mean titer of the *GFP* dsRNA control samples. The results of two independent experiments with 4 replicates each were then combined after normalization. RNA was extracted to determine levels of gene knockdown and virus RNA levels by real-time RT-PCR compared to the *Rs86* and *GFP* controls.

### Determination of tick mRNA levels by quantitative real-time RT-PCR

The expression of selected genes was characterized using total RNA extracted from tick cells or individual female guts and salivary glands. All samples were confirmed as infected or uninfected by real-time PCR analysis of *A*. *phagocytophilum msp4* DNA in tick cells, guts and salivary glands. Real-time RT-PCR was performed on RNA samples using gene-specific oligonucleotide primers for *I*. *scapularis* genes encoding Tudor-SN (XP_002415051; TUD5: 5‘-TGGAGGAGATGCTGACACTG-3‘ and TUD3: 5‘-TCCTTCGAGTCGTCCTCTGT-3‘), AgoA (XM_002401346; Ago15: 5‘-AAGGGTGATCAGAAGCTCCA-3‘ and Ago13: 5‘-GTTCCGACCAAGGACGACTA-3‘) and SUB [27] and the iScript One-Step RT-PCR Kit with SYBR Green and the CFX96 Touch Real-Time PCR Detection System (Bio-Rad, Hercules, CA, USA). A dissociation curve was run at the end of the reaction to ensure that only one amplicon was formed and that the amplicons denatured consistently in the same temperature range for every sample. The mRNA levels were normalized against tick 16S rRNA and cyclophilin as described previously using the genNorm method (ddCT method as implemented by Bio-Rad iQ5 Standard Edition, Version 2.0) [27]. Normalized Ct values were compared between test dsRNA-treated ticks and control ticks treated with *Rs86* dsRNA, between infected and uninfected ticks or between tick feeding days 0, 1 or 3 and day 5 by Student's t-test with unequal variance (P = 0.05).

For analysis of *Tudor-SN* mRNA levels in different tick developmental stages and tissues, total RNA was extracted from *I*. *scapularis* eggs (three batches of approximately 500 eggs each), fed and unfed larvae (three pools of 50 larvae each), fed and unfed nymphs (three pools of 15 nymphs each), and fed and unfed male and female adult tick tissues (4 ticks each) were used for real-time RT-PCR as described above but normalizing against tick *cyclophilin* and *ribosomal protein S4* (DQ066214) using oligonucleotide primers rsp4-F: 5’-GGTGAAGAAGATTGTCAAGCAGAG-3’ and rsp4-R: 5‘-TGAAGCCAGCAGGGTAGTTTG-3’ [23]. Normalized Ct values were compared between unfed and fed ticks by Student's t-test with unequal variance (P = 0.05).

### Determination of *A*. *phagocytophilum* infection by quantitative real-time PCR

*A*. *phagocytophilum* DNA levels were characterized by *msp4* real-time PCR normalizing against tick *16S rDNA* as described previously [27]. Normalized Ct values were compared between groups by Student's t-test with unequal variance (P = 0.05).

### Determination of LGTV replication by quantitative real-time RT-PCR

Total RNA was extracted using an RNeasy Mini kit (QIAGEN, Manchester, UK) according to the manufacturer’s instructions. cDNA was prepared from 1 μg RNA per reaction using the High Capacity cDNA Reverse Transcription Kit (Life Technologies, Paisley, UK). Quantitative real-time PCR was performed on a ViiA 7 Real-Time PCR System (Life Technologies, Paisley, UK) using the FastStart Universal SYBR Master (Roche Applied Science, Burgess Hill, UK). Actin was used as a housekeeping gene for normalization as described previously [51]. Relative LGTV RNA levels were then normalized to the *GFP* dsRNA control by dividing the value of each sample by the mean titer of the *GFP* dsRNA control samples. Two independent experiments with 4 replicates each were then combined after normalization and statistical significance was determined by comparing *Tudor-SN* dsRNA-treated cells to cells incubated with the *GFP* dsRNA control by two-way ANOVA with multiple comparisons (Fisher’s LSD test; P = 0.05).
